# A novel EGFR-TKI inhibitor (cAMP-H_3_BO_3_complex) combined with thermal therapy is a promising strategy to improve lung cancer treatment outcomes

**DOI:** 10.18632/oncotarget.17628

**Published:** 2017-05-05

**Authors:** Yongpeng Tong, Chunliu Huang, Junfang Zhang

**Affiliations:** ^1^ College of Physics and Energy, Shenzhen University, Shenzhen, 518060, China; ^2^ School of Medicine, Sun Yat-Sen University, Guangzhou, 510080, China; ^3^ School of Medicine, Shenzhen University, Shenzhen Second People's Hospital, Shenzhen, 518060, China

**Keywords:** cAMP-H_3_BO_3_ complex, EGFR-TKI, thermal therapy, NSCLC

## Abstract

**Purpose:**

Although EGFR-TKIs (epidermal growth factor receptor tyrosine kinase inhibitors) induce favorable responses as first-line non-small cell lung cancer treatments, drug resistance remains a serious problem. Meanwhile, thermal therapy also shows promise as a cancer therapy strategy. Here we combine a novel EGFR-TKI treatment with thermal therapy to improve lung cancer treatment outcomes.

**Results:**

The results suggest that the cAMP-H_3_BO_3_ complex effectively inhibits EGFR auto-phosphorylation, while inducing apoptosis and cell cycle arrest *in vitro*. Compared to the negative control, tumor growth was significantly suppressed in mice treated with oxidative phosphorylation uncoupler thyroxine sodium and either cAMP-H_3_BO_3_ complex or cAMP-H_3_BO_3_ complex (*P* < 0.05). Moreover, the body temperature increase induced by treatment with thyroxine sodium inhibited tumor growth. Immunohistochemical analyses showed that A549 cell apoptosis was significantly higher in the cAMP-H_3_BO_3_ complex plus thyroxine sodium treatment group than in the other groups. Moreover,Ca^2+^ content analysis showed that the Ca^2+^ content of tumor tissue was significantly higher in the cAMP-H_3_BO_3_ complex plus thyroxine sodium treatment group than in other groups.

**Materials and Methods:**

Inhibition of EGFR auto-phosphorylation by cAMP and cAMP-H_3_BO_3_ complex was studied using autoradiography and western blot. The antitumor activity of the novel EGFR inhibitor (cAMP-H_3_BO_3_ complex) with or without an oxidative phosphorylation uncoupler (thyroxine sodium) was investigated *in vitro* and in a nude mouse xenograft lung cancer model incorporating human A549 cells.

**Conclusions:**

cAMP-H_3_BO_3_ complex is a novel EGFR-TKI. Combination therapy using cAMP-H_3_BO_3_ with thyroxine sodium-induced thermal therapy may improve lung cancer treatment outcomes.

## INTRODUCTION

Lung cancer is the most common cause of cancer deaths worldwide. Because prognosis is often poor, new treatment strategies are urgently needed [1, 2]. Previous studies have shown that epidermal growth factor receptor (EGFR)-mediated cellular signaling plays a key role in numerous signaling pathways [3, 4]. Furthermore, activation of downstream signal transduction pathways (MEK/ERK) by EGFR has been shown to inhibit apoptosis in normal keratinocytes by increasing CyclinD1 and Bcl-2 levels, promoting formation of blood vessels and enhancing tumor survival [4]. Patients with non-small cell lung cancer (NSCLC) usually possess specific mutations in the tyrosine kinase domain of the EGFR gene, the clinical target of EGFR tyrosine kinase inhibitor (EGFR-TKI) therapy [4, 5]. EGFR-TKIs such as gefitinib, erlotiniband afatinibare currently used in the clinical treatment of cancer [5, 6] and compared to standard chemotherapy treatments, first-line treatment with EGFR-TKIs induces favorable anti-tumor responses. However, development of tumor resistance remains a serious problem that necessitates ongoing development of novel EGFR inhibitors [7].

Tyrosine kinase (TK) signaling has garnered much interest in recent years, principally in cancer research, due to its demonstrable success as a point of action for precision targeting of drugs acting upon critical pathogenic drivers [8]. Under normal conditions, tyrosine phosphorylation acts as a rapid on-off switch in cells. It is employed by cellular signaling pathways to regulate growth, migration, adhesion, differentiation and survival, as well as proliferation signals promoting tumor development [9–11]. While some cAMP-analogue compounds have been used as EGFR-TKIs with good efficacy [11], most lose effectiveness due to drug resistance, which may result from recognition of novel molecules (such as: 8-chloro-cAMP) by the immune system. Here we study a novel EGFR inhibitor (cAMP-H_3_BO_3_ complex) which can inhibit EGFR auto-phosphorylation with high efficacy. Furthermore cAMP-H_3_BO_3_ complex is composed of two small molecules, each with low toxicity and bound together by an ionic bond instead of a covalent bond. For this reason, the complex should be less likely to promote drug resistance, as low toxic small molecules tend to be non-immunogenic.

Meanwhile, thermal therapy to eradicate tumors has garnered more and more attention from researchers [12, 13]. One new idea under consideration entails the use of high temperature to manipulate tumor growth. Elucidation of the mechanisms responsible for killing of abnormal cells induced by temperature change can be estimated from direct measurements during tumorigenesis [14]. Cells normally radiate heat due to activities within mitochondria. These organelles generate ATP through oxidative phosphorylation (OXPHOS) through controlled substrate degradation and oxygen consumption. However, not all energy production is coupled to ATP synthase; some energy is dissipated as heat, accounting for 20–30% of the basal metabolic rate [15]. This nonproductive energy leak, termed “mitochondrial uncoupling,” plays an important role in reprogramming cancer cell metabolism. Therefore, in cancer cells, regulation of mitochondrial dysfunction and associated cellular bioenergetics shows promise as a target for chemotherapeutic drug development [16].

One goal of this study focused on expanding our understanding of cellular heat generation induced by certain OXPHOS uncoupling compounds (thyroid hormones, etc.) [17]. Due to their higher energy demands, the OXPHOS activity of cancer cells may greatly exceed that of noncancerous cells. Consequently, uncoupling of OXPHOS in cancer cells may result in higher thermal release and apoptosis than in noncancerous cells [18]. In lieu of extraneously applied heat, use of an uncoupler may be a better way to carry out thermal therapy from within cells [19], achieving self-controlled drug administration with maintenance of safer temperatures during tumor treatment. Based on these concepts, the model system described herein was developed and should help us gain insight regarding the optimal temperature for cancer treatment. Furthermore, both cAMP and heat induce cell apoptosis by inducing calcium influx to achieve antitumor effects. Calcium influx can be measured and thus may provide additional insights into a mechanism to guide optimization of apoptotic effects.

In this study, we developed a novel EGFR-TKI inhibitor (cAMP-H_3_BO_3_ complex), which significantly inhibits EGFR auto-phosphorylation and induces lung cancer cell apoptosis. By increasing body temperature using an OXPHOS uncoupling agent (thyroxine sodium) in combination with EGFR pathway inhibition, we achieved significant tumor growth inhibition. This strategy is a promising approach for improvement of lung cancer outcomes.

## RESULTS

### cAMP-H_3_BO_3_ complex synthesis

**Figure 1:**
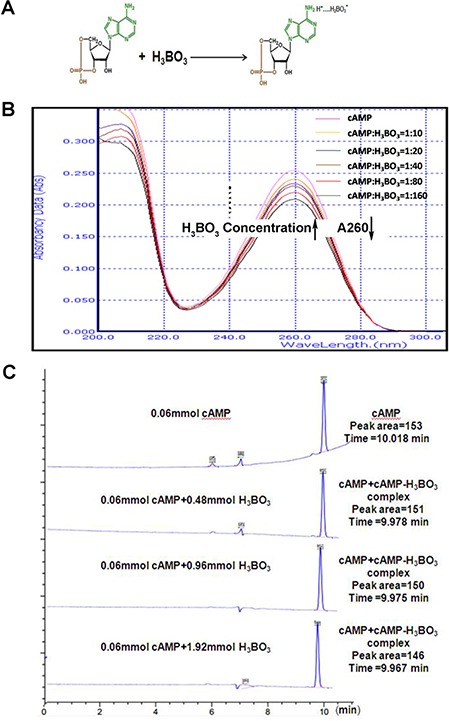
cAMP-H_3_BO_3_ complex synthesis (**A**) the model of cAMP-H_3_BO_3_ complex formation; (**B**) UV absorption spectra for various concentrations of H_3_BO_3_ solution (0.06–1.88 mM) and a concentration cAMP solution (0.006 mM) are shown; (**C**) the typical HPLC spectra of 0.06 mM cAMP with various H_3_BO_3_ concentrations. Note that with increasing H_3_BO_3_ concentration, the retention time of the main peak (9.9–10.1) is slightly decreased and peak area is also decreased.

It is well known that H_3_BO_3_ can react with multiple hydroxyl compounds (sugars, nucleotides, etc.) to form complexes with high stability constants. Formation of such complexes often facilitates their electrophoretic separation and characterization [20]. Figure 1A illustrates a model describing how cAMP and H_3_BO_3_ form a complex. The UV absorption spectra for various H_3_BO_3_ solution concentrations for a single cAMP solution concentration are shown in Figure 1B. The magnitude of the absorption peak of cAMP (0.006 mM) at 260 nm decreased with increasing H_3_BO_3_ concentration (0.06–1.88 mM). To further confirm this result, we analyzed the typical HPLC spectra for 0.06mM cAMP in the presence of various H_3_BO_3_ concentrations (Figure 1C). As H_3_BO_3_ concentrations increased, the retention time of the main peak (9.9–10.1 min) slightly decreased and the peak area decreased as well. Because thecAMP-H_3_BO_3_ complex contains more OH groups than does H_3_BO_3_, it is more easily eluted by methanol and exhibits a slightly lower HPLC retention time, thus demonstrating formation of the cAMP-H_3_BO_3_ complex. This result agrees with previous data for cAMP-H_3_BO_3_ where by capillary electrophoresis mobility changed with changing boric acid concentration [14]. Such mobility changes reflect formation of the complex form, taking into account the fact that the basic amino cAMP base can bind to the hydrogen ion of boric acid to form a salt. This observation also explains the effect of various boric acid concentrations on spectral changes observed for cAMP, the UV-absorbing base component of the resulting acid-base complex.

### Treatment with cAMP-H_3_BO_3_ complex induces apoptosis and cell cycle arrest

**Figure 2:**
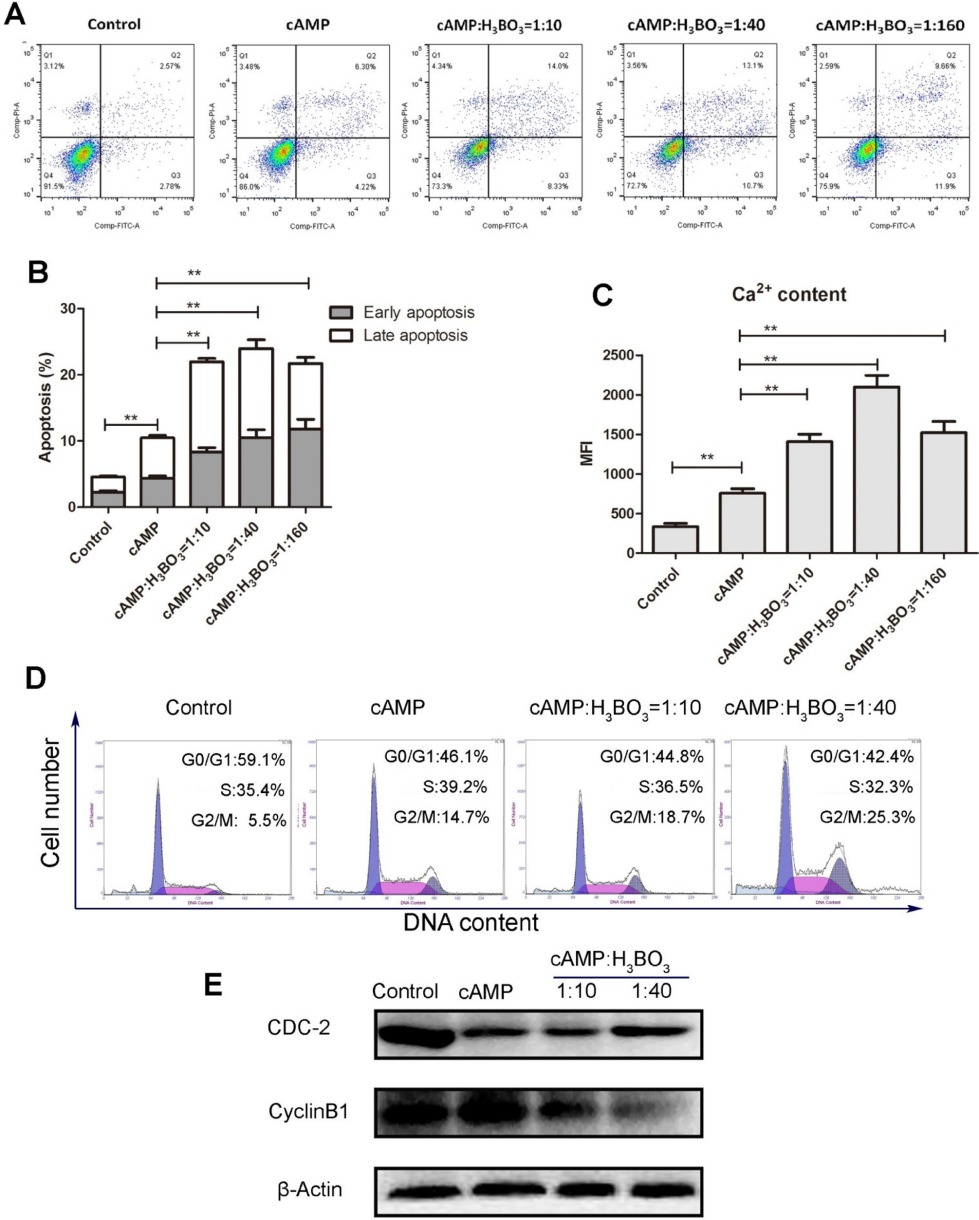
cAMP-H_3_BO_3_ complex induce apoptosis and cell cycle arrest cAMP-H_3_BO_3_ complex preparations containing various ratios of constituent compounds (cAMP:H_3_BO_3_ = 1:10;1:40;1:160) were used to treat A549 cells in the concentration of 200 μM for 48 hrs. (**A**) cell apoptosis were analysed by flow cytometry; (**B**) statistical analysis the data from A; (**C**) Ca^2+^ content were analysed by flow cytometry; (**D**) cell cycle arrest were analysed by flow cytometry; (**E**) G2/M phase related regulatory protein (cyclinB1 and CDC-2) were detected by western blotting. Statistical analysis was performed using one-way ANOVA and Tukey HSD post hoc test to compare all pairs of data; *p* ≤ 0.05 was considered statistically significant.

Next, we investigated the anti-tumor effect of the cAMP-H_3_BO_3_ complex. For complex formation, solutions containing various cAMP: H_3_BO_3_ ratios (cAMP: H_3_BO_3_ = 1:10, 1:40, 1:160) were prepared. To these solutions, thyroxine sodium was added to achieve either a high (200 μM) or low (100 μM) uncoupler concentration. The solutions were then added to A549 cells and incubated for 48 hours. The result was shown in Figure 2. As shown in Figure 2A and 2B, cAMP alone significantly induced more cell apoptosis than the cell without cAMP (negative control) did, while the cAMP-H_3_BO_3_ complex enhanced significantly apoptosis than did cAMP alone (Figure 2B). Consistent results was also showed in other cancer cell lines (Supplementary Figure 1A) Meanwhile, cAMP alone significantly increased Ca^2+^ content more than observed for the control, while cAMP-H_3_BO_3_ complex significantly increased Ca^2+^ level more than observed for cAMP treatment alone(Figure 2C). We also observed cell cycle arrest in the G2/M phase after cAMP-H_3_BO_3_ complex treatment (Figure 2D); levels of relevant regulatory mitotic proteins (cyclinB1 and CDC-2) were significantly lower than for the control as viewed by western blot (Figure 2E).

### cAMP-H_3_BO_3_ complex inhibit EGFR auto-phosphorylation

**Figure 3:**
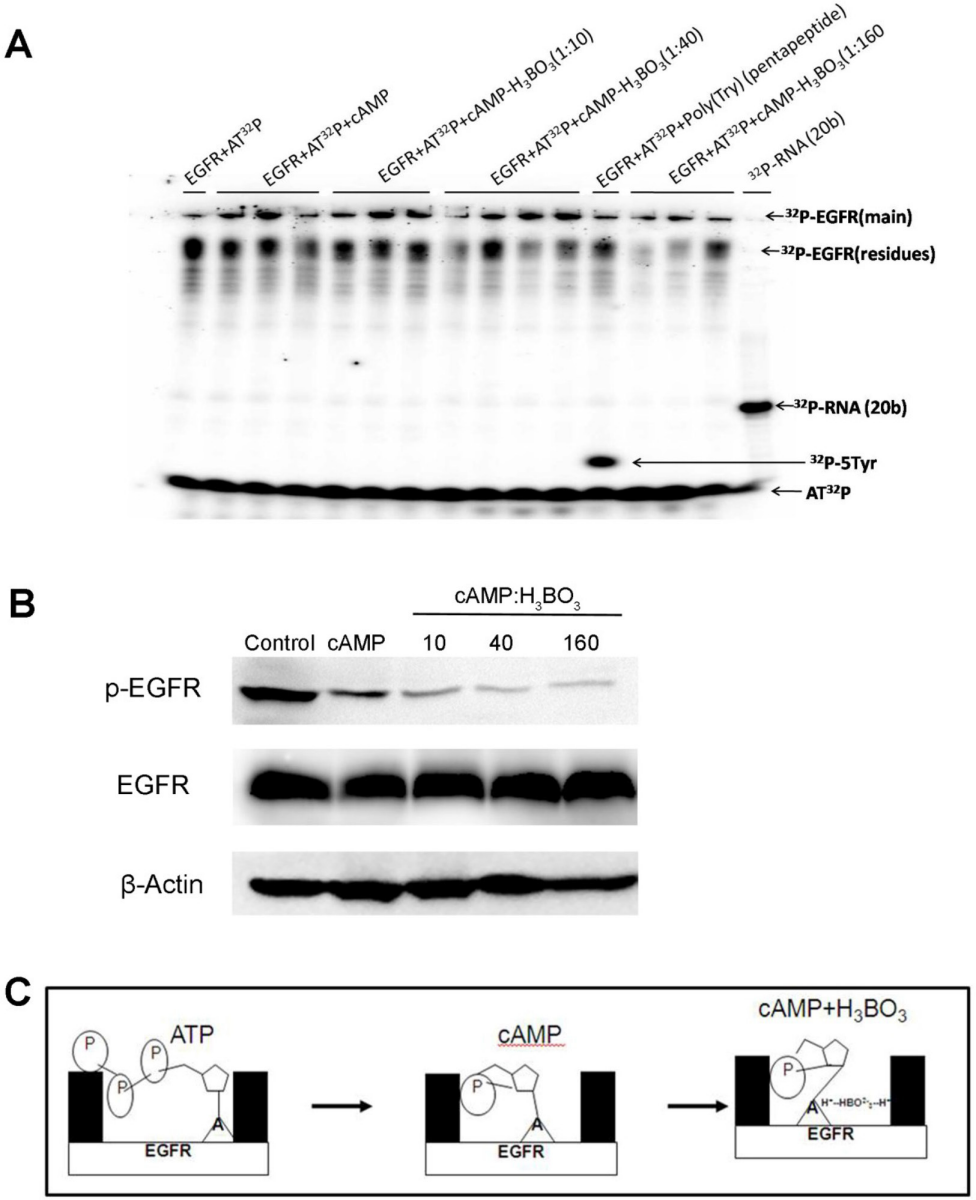
cAMP-H_3_BO_3_ complex inhibit EGFR phosphorylation (**A**) The inhibition of auto-phosphorylation of EGFR by the cAMP-H_3_BO_3_ complex. ^32^P-ATP autoradiography study showing inhibition of auto-phosphorylation of EGFR bythecAMP-H_3_BO_3_ complex; (**B**) The inhibition of auto-phosphorylation of EGFR by the cAMP-H_3_BO_3_ complex in A549 cells; (**C**) The pattern of cAMP-H_3_BO_3_ complex inhibition of EGFRauto-phosphorylation.

The inhibition of auto-phosphorylation of EGFR by cAMP and cAMP-H_3_BO_3_ complex was also investigated. As shown in Figure 3A, EGFR auto-phosphorylation products labeled with ^32^P are shown in different lanes after they absorbed ^32^P from ^32^P-labeled ATP in the reaction buffer. Compared to the control, EGFR alone (lane 1) and cAMP+EGFR (lanes 2–4) exhibited significantly lower levels of ^32^P-labeled EGFR auto-phosphorylation products. Furthermore, cAMP-H_3_BO_3_ complex+EGFR (lanes 5–7) generated even lower amounts of ^32^P-labeled EGFR auto-phosphorylation products than did cAMP+EGFR (lane 2) and EGFR auto-phosphorylation product levels decreased as either cAMP or cAMP-H_3_BO_3_ complex concentrations increased. Quantification and statistic analysis was shown in Supplementary Figure 1B. To further confirm this result, we treated the lung cancer cell line (A549) with cAMP-H_3_BO_3_ complex and found that the phosphorylation of EGFR was significantly decreased compared to that achieved with cAMP alone (Figure 3B). Thus, this result demonstrates that cAMP-H_3_BO_3_ complex most effectively inhibits EGFR auto-phosphorylation of treatments tested.

Taking into account that the basic amino group in the cAMP base and the basic amino acid in the ATP-pocket of EGFR can each bond with boric acid through ionic and hydrogen bonds, the cAMP-H_3_BO_3_ complex should form a more stable complex with the EGFR ATP-binding pocket than would cAMP alone. The scheme, illustrated in Figure 3C, shows that cAMP-H_3_BO_3_ complex has a greater ability to compete with and block ATP binding to the ATP-binding site than does cAMP.

### cAMP-H_3_BO_3_ complex plus thyroxine sodium inhibited lung cancer growth in a nude mouse xenograft model

The NSCLC cell line (A549) nude mouse xenograft model was used to investigate tumor growth inhibition. Three groups of five mice per group received oral treatments as follows: group A, the negative control group, received deionized drinking water; group B received deionized drinking water containing cAMP-H_3_BO_3_ complex; group C, received deionized drinking water containing thyroxine sodium+cAMP-H_3_BO_3_ complex. After 4 weeks of drug treatments, tumor tissue weight, survival proportions, body weight, body average temperature and tumor tissue Ca^2+^ content were recorded for mice in the three groups.

**Figure 4:**
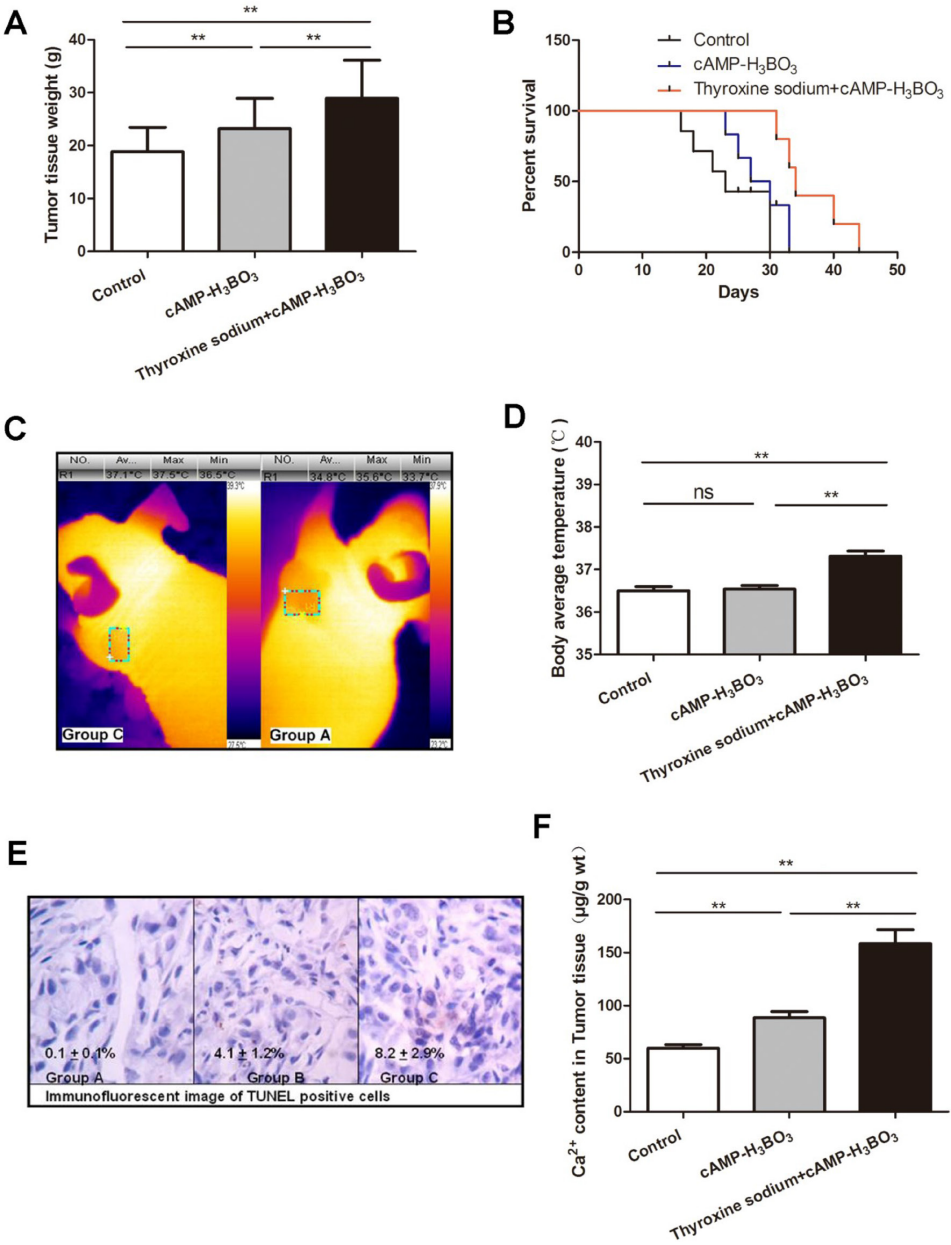
Combined cAMP-H_3_BO_3_ complex and thyroxine sodium inhibit tumor growth in nude mouse xenograft model The NSCLC cell line (A549) nude mouse xenograft model was used to investigate tumor growth inhibition. Three groups of five mice each (*n* = 5) were fed treatments in drinking water (deionized water):control group received deionized water (negative control); cAMP-H_3_BO_3_ group received deionized water+cAMP-H_3_BO_3_ complex solution; Thyroxine sodium+cAMP-H_3_BO_3_ group received deionized water+Thyroxine sodium+cAMP-H_3_BO_3_ complex solution. Tumor tissue weight (**A**), survival proportion (**B**), The temperature of tumor areas (**C**), body average temperature (**D**), TUNEL showing slides of apoptotic cells (**E**) and Ca^2+^ content in tumor tissue of the 3 groups (**F**) are shown. All treatments were orally administered for four weeks. A, Tumor tissue weight (g); B, Survival proportions; C, Body weight (g); D, Body average temperature (°C); E, The typical tunel assay of tumor tissue sections; F, Ca^2+^ content in Tumor tissue (μg/g wt). Statistical analysis was performed using one-way ANOVA and Tukey HSD post hoc test to compare all pairs of the data and *p* ≤ 0.05 was considered statistically significant.

Tumor tissue weights of both groups B and C were significantly lower than that of group A, while group C weights were significantly lower than those of group B (Figure 4A). Survival proportions of both B and C groups were significantly higher than for group A, while group C survival significantly exceeded that of group B (Figure 4B). No statistically significant changes in body weight among the three groups of mice were observed during the course of the study (Figure 4C) (Supplementary Figure 1C). The temperature of tumor areas in group C is significantly higher than that in group A (Figure 4C, Supplementary Figure 1E). The average body temperature of group C was significantly higher than that of groups A and B (Figure 4D, Supplementary Figure 1D). From Figure 4E it is clear that counts of TUNEL-positive cells (apoptotic cells) were significantly higher in group C than in groups B or A. Tumor Ca^2+^ content was also significantly higher in group C vs. groups B or A (Figure 4F). Therefore, increased apoptosis may serve as the major mechanism underlying tumor growth inhibition after treatment using combination therapy including an OXPHOS uncoupling agent with an EGFR-TKI.

These results suggest that the cAMP-H_3_BO_3_ complex can inhibit tumor growth significantly. Meanwhile, increased body temperature induced by the OXPHOS uncoupler thyroxine sodium may also help to inhibit tumor growth. In this study, we observed that the use of both a thermal therapeutic agent and an EGRF-TKI together was more effective than either agent alone for inducing apoptosis in A549 cells. This result was further supported by the higher apoptosis rate and higher calcium influx observed in cells from group C vs. A or B mice.

## DISCUSSION

EGFR-TKI therapy achieves favorable clinical outcomes for patients with non-small cell lung cancer (NSCLC) [21]. This is due, in part, to the regulatory function of EGFR. EGFR dimerization stimulates its own intracellular protein tyrosine kinase activity, which then triggers cellular signaling that may lead to proliferation. Because TKIs reversibly bind to EGFR, they preferentially inhibit ATP binding to the EGFR tyrosine kinase domain, thereby halting cell proliferation and promoting cell death [22]. Three generations of EGFR-TKIs are currently in use and exploit distinct mechanisms of action. First generation TKIs (e.g., Erlotinib) have been shown to substantially improve response rates to standard chemotherapy regimens in patients possessing a sensitizing EGFR mutation. Second-generation EGFR-TKIs (e.g., Afatinib), which irreversibly bind to the tyrosine kinase of EGFR, are used as first-line treatments for advanced NSCLC harboring activating EGFR mutations; however, acquired resistance remains an intractable problem [23, 24]. Osimertinib (Tagrisso), a third generation TKI which forms an irreversible covalent bond to EGFR, specifically targets the T790M mutation, resulting in prevention of EGFR resistance mutations. Although treatment is generally well tolerated, notable side effects include diarrhea, stomatitis, skin and nail changes, shortness of breath, pneumonitis and bone marrow suppression [25, 26]. For these reasons, research to develop new highly effective novel EGFR-TKIs with low toxicity are urgently needed. Here, a novel candidate EGFR-TKI, cAMP-H_3_BO_3_ complex, is investigated for its ability to reversibly bind to the EGFR ATP-binding site to prevent EGFR signal transduction and subsequently inhibit cancer cell growth.

It is well known that mitochondria are the center of OXPHOS activity and energy metabolism. They also play a very important role in the regulation of cellular metabolism, calcium homeostasis, redox signaling and cell fate [27]. Indeed, emerging evidence indicates that mitochondrial dysfunction is the defining characteristic of nearly all types of cancers. Therefore, targeting of mitochondria is a promising therapeutic cancer treatment strategy [28]. Thyroxine sodium, which functions as a thyroid hormone, acts within mitochondria of each cell of the body, increasing the basal metabolic rate and leading to heat generation with occasional induction of higher body temperature [28]. Indeed, thyroxine sodium administered orally to mice can induce marked body temperature changes, as confirmed in our experiments. Therefore, thyroxine sodium is a promising anticancer thermal treatment agent.

Another heat-generating treatment, radiofrequency ablation (RFA), is a minimally invasive form of therapy that uses external sources of electrical energy and heat to kill cancer cells. Ablation techniques generate inflammatory immune responses and tissue disruption to achieve their effects. Additional anticancer thermal therapies using extraneous heat sources are popular nowadays and include microwave (MW) irradiation and magnetic resonance (MR) cancer treatments [29]. Such thermal therapeutic modalities focus heat produced outside the body onto tumor tissues, but would be ineffective for treating non-localized cancers such as leukemia. Conversely, endogenous heat generated within cancer cells may be advantageous for treating cancer cells disseminated throughout the body. Moreover, this method specifically targets cells that primarily obtain energy from ATP generation via OXPHOS, a more important source of energy for tumor cells than for noncancerous cells. Therefore, the careful selection of an effective OXPHOS uncoupler for delivery of thermal therapy to cancer cells may be a first step toward development of an effective thermal therapy to treat cancer.

## MATERIALS AND METHODS

### cAMP-H_3_BO_3_ complex synthesis

10 μl 1.8 mM cAMP (Shanghai Yuanye Bio-Technology Co., Ltd., China, > 98% purity) solution was mixed with various volumes of 90 mM H_3_BO_3_(2 μl, 4 μl, 8 μl, 16 μl, 32 μl or 64 μl)(Sangon Biotech Co., Ltd, China, > 98% purity) and adjusted to pH 7.0 using NaOH(Sangon Biotech Co., Ltd, > 98% purity). The mixtures were incubated at room temperature for 5min to allow thecAMP-H_3_BO_3_ complexto form. Solutions containing cAMP-H_3_BO_3_ complex were diluted to a volume of 3mlusing deionized water. Next, solutions containing cAMP-H_3_BO_3_ complex(cAMP, 0.006 mM and H_3_BO_3_, 0.06–1.88 mM) were detected by UV absorption spectrophotometry (UV 754N, China) using absorption in the wave length range of 200–300 nm.

### HPLC spectral analysis

An Agilent 6120 LC/MS system (a triple quadrupole mass spectrometer) was used to analyze solutions containing 5X (X = A, G, C, T, U oligonucleotides) with ATP (designated 5X+ATP) and with or without EGFR. An Agilent Single Quadrupole LC/MS (G6120, Agilent, CA, USA) with Agilent Mass Hunter Walkup Software for LC/MS and LC systems was used for data acquisition. UV detection was performed at 260 nm. Analytes were separated using an Ecosil C18 column (250 mm × 4.6 mm; Guangzhou Lubex Biological Technology Co., Ltd., China). The mobile phase consisted of a mixture of (A) 0.1% formic acid (LC/MS grade) and (B) methanol (LC/MS grade). A gradient elution method was used (Time: 0-2-16-20-25-25.1-30 min using solvent B: 0%-0%-30%-90%-90%-0%-0%). Milli-Q water was used in all experiments.

### Autoradiography study of the inhibition of EGFR auto-phosphorylation bythecAMP-H_3_BO_3_ complex

In order to examine the inhibition of EGFR auto-phosphorylation by cAMP and cAMP-H_3_BO_3_ complex, a ^32^P-ATP autoradiographic study was conducted using polyacrylamide gel electrophoresis (PAGE). Reactions contained 2 μl of 0.52 mg/ml human EGFR/HER1/ErbB1 (aa668-1210)(the recombinant human EGFR/GST chimera consisting of 780 amino acids with a calculated molecular mass of 89.1 kDa was purchased from Sino Biological Inc., P.R. China, > 85% purity), 1 μl [γ-^32^P]-ATP (3000 Ci/mmol, 10 mCi/ml, PerkinElmer Co., CA, USA) and 1.2 μl of 10X T4 PNK reaction buffer A (from Sino Biological Inc., P.R. China), To this mix were added various volumes of inhibitors (2 μl, 4 μl or 8 μl of 5 μM cAMP (Sangon Biotech Co., Ltd, > 98% purity) or (1 μl, 2 μl or 3 μl of 5 mMH_3_BO_3_ with 2 μl of 5 μM cAMP) or no inhibitor (control). Each reaction solution was adjusted to a total final volume of 12 μl with deionized water.

After incubation for 24 h at 37°C, 5 μl of each reaction solution was electrophoresed on denaturing 7M urea PAGE gels (15 cm × 15 cm) using 1X TBE running buffer and 10W of power for 2 h at room temperature. After separation, gels were covered with a phosphor screen to collect photo-stimulated luminescence from the imaging plate after exposure to γ radiation from the ^32^P-gel for 3h. Next, the phosphor screen was read using a Typhoon FLA 7000 laser scanner (GE Healthcare, UK). Lane 1 shows EGFR+^32^P-ATP alone. Lanes 2-4 show the scanning map of EGFR+^32^P-ATP+cAMPreaction mixture (containing various cAMP concentrations). Lanes 5-7 show the scanning map of the EGFR+^32^P-ATP+cAMP+H_3_BO_3_ reaction mixture containing various H_3_BO_3_ concentrations. Lane (8) shows the scanning map of the enzyme activity control (Poly(Tyr)---5Tyr(pentapeptide)) marker. Lane 9 shows the scanning map of autoradiography control marker(^32^P-RNA, 20bases in length) obtained by reaction of RNA (20bases in length) with T4 PNK in the presence of [γ-^32^P]-ATP. The autoradiography result is shown in Figure 2.

### Anti-tumor animal experiment

A nude mouse xenograft model was developed that was xenografted with a human lung cancer cell line, NSCLC A549, which possesses wild type EGFR and EGFR expression. This model was developed by Guangdong Animal Experimental Center, which supplied the mice for the experiments in this study and housed them until tumor xenografts reached 5 to 6 mm in diameter then shipped the mice to our laboratory. All supplied mice were female and 19-20g in weight. Mice were randomly divided into 3 groups that received different drinking regimens: deionized water for negative control group(A), deionized water+cAMP-H_3_BO_3_ complex solution for group (B) and deionized water+thyroxine sodium+cAMP-H_3_BO_3_ complex solution for group (C). Thyroxine sodium was purchased from Sangon Biotech Co., Ltd. (> 98% purity). All treatments were administered for four weeks.

All treatments were administered orally and the final solutions were adjusted to pH 7.0 with NaOH before administering the treatments. The cAMP concentration was 1.6 mg/ml and the H_3_BO_3_ concentration was 16 mg/ml in the cAMP-H_3_BO_3_ complex (group B). The cAMP concentration was 1.6 mg/ml, H_3_BO_3_ concentration was 16 mg/ml and the thyroxine sodium concentration was 0.010 mg/ml in the thyroxine sodium+cAMP-H_3_BO_3_ complex drinking solution (group C). The drinking water and food were made freely available to all groups. The dose of drug solution was kept at 0.20–0.30 ml/day per mouse.

After four weeks of treatment, mouse body weights and body temperatures were recorded (Figure 4). All mouse body temperatures were determined using a Thermal Imager (CEM DT-982). Typical temperature mapping results are shown in Figure 3 and Figure 4. All tumor cell xenografts were resected and weighed and are compared in Figure 4. Next, portions of tumor tissues were subjected to immunohistochemical analysis using a terminal deoxynucleotidyl transferase (TdT) dUTP nick end labeling (TUNEL) assay to detect tumor cell apoptosis. Tumor tissue samples were assayed for Ca^+2^ contents using inductively coupled plasma mass spectrometry (ICP-MS) and the results are recorded in Figure 4. Representative results of immunohistochemical analysis are shown in Figure 4.

### Non-contact body temperature measurement

Each day at 9 am the average body temperatures of mice were measured 30 min after feeding using a Thermal Imager (CEM DT-982).

### Immunohistochemistry process

Frozen tumor tissues were sliced into sections and 5 μM thick paraffin sections were subjected to TUNEL reaction using an In situ Apoptosis Detection Kit (Takara Bio, Inc.) according to the manufacturer’s instructions. Samples were observed under a microscope (Olympus, Japan). The details of the process were published previously [26].

### ICP-MS analysis

0.2 g of each grated tumor tissue sample was added to a polytetrafluoroethylene (PTFE) digestion container. A volume of 8ml of H_3_BO_3_ was added to each sample then samples were incubated for 30 min at room temperature. The specimens were digested in a Microwave Digestion System (Ethos One; Milestone, Italy). After digestion, each solution was diluted with deionized water to a total volume of 50 ml. Further analyses were conducted using ICP-MS (NexIon300X; PerkinElmer, USA). The detailed procedure is based on our previous work [25].

### Statistical analysis

Statistical analysis was performed using one-way ANOVA and Tukey HSD post hoc test to compare all pairs of the data and *p* ≤ 0.05 was considered statistically significant.

## CONCLUSIONS

Our current study revealed that the cAMP-H_3_BO_3_ complex is a novel EGFR inhibitor which can inhibit tumor growth. Use of an OXPHOS uncoupling agent together with an EGFR inhibitor is a good combination to achieve anti-tumor thermal therapy, especially in body regions where less controllable extraneous heating therapies are contraindicated. In such cases, the uncoupler thyroxine may be recommended for induction of apoptosis of tumor cells growing in body regions of particular concern (e.g., growing in the neck region), where patients’ thermoregulatory responses cannot reliably compensate to maintain safe temperature levels during MW or MR therapy.

## SUPPLEMENTARY MATERIALS FIGURES AND TABLES



## Abbreviations

EGFR: epidermal growth factor receptor
NSCLC: non-small cell lung cancer
EGFR-TKI: EGFR tyrosine kinase inhibitor
