# Supplementary material for: A novel EGFR-TKI inhibitor (cAMP-H3BO3complex) combined with thermal therapy is a promising strategy to improve lung cancer treatment outcomes
Source: Oncotarget. 2017 May 5;8(34):56327–37. doi: 10.18632/oncotarget.17628 (PMC5593564; doi:10.18632/oncotarget.17628)
Supplement: Supplementary file 1 [file oncotarget-08-56327-s001.pdf]

A novel EGFR-TKI inhibitor (cAMP-H_3BO_3complex) combined with thermal therapy is a promising strategy to improve lung cancer treatment outcomes

Supplementary Materials

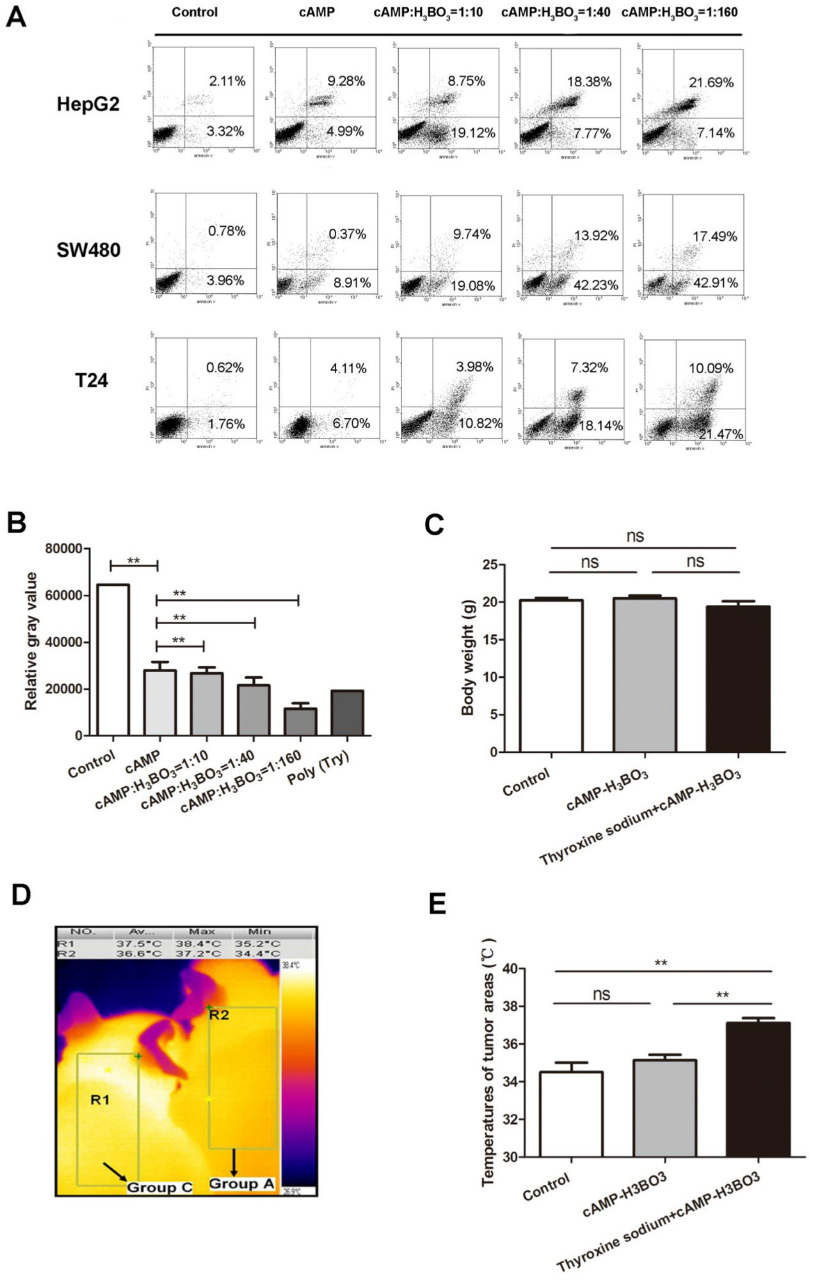

Supplementary Figure 1: (**A**) cAMP-H_3BO_3 complex with different rate (cAMP: H_3BO_3 =1:10;1:40;1:160) were used to treat liver cancer (HepG2), intestinal cancer(SW480), Bladder cancer (T24). with high concentration (200 μM) for 48 hours, cell apoptosis were analysed by flow cytometry; (**B**) Quantification and statistic analysis of Figure 3A; NSCLC cell line (A549) nude mouse xenograft model were used to investigate the tumor growth inhibition. Three groups ($n = 5$): control group, drinking the deionized water for negative control; cAMP-H_3BO_3 group, drinking the deionized water + cAMP-H_3BO_3 complex solution; Thyroxine sodium+cAMP-H_3BO_3 group, drinking the deionized water + Thyroxine sodium+cAMP-H_3BO_3 complex solution. The drugs were orally administered for 4 weeks. (**C**) the body weight of the 3 groups; (**D**) the body temperature of Group A and Group B; (**E**) the temperature of tumor areas in the 3 groups.
